# Improving Home-Based Scoliosis Therapy: Findings From a Web-Based Survey

**DOI:** 10.2196/46217

**Published:** 2023-08-04

**Authors:** Florian Günther, Fabian Schober, Sandra Hunger, Julia Schellnock, Steffen Derlien, Stefan Schleifenbaum, Welf-Guntram Drossel, Christoph-Eckhard Heyde

**Affiliations:** 1 Department of Biomechatronics Fraunhofer Institute for Machine Tools and Forming Technology IWU Dresden Germany; 2 Center for Research on Musculoskeletal Systems (ZESBO) Faculty of Medicine Hospital of Orthopaedic Surgery, Traumatology and Plastic Surgery, University of Leipzig Medical Center Leipzig Germany; 3 Hörmann Vehicle Engineering GmbH Dresden Germany; 4 Scientific Field Functional Integration and System Integration Fraunhofer Institute for Machine Tools and Forming Technology IWU Dresden Germany; 5 Institute for Physical and Rehabilitative Medicine University Hospital Jena Jena Germany; 6 Professorship Adaptronics and Lightweight Design Technical University Chemnitz Chemnitz Germany; 7 Hospital of Orthopaedic Surgery, Traumatology and Plastic Surgery University of Leipzig Medical Center Leipzig Germany

**Keywords:** scoliosis therapy, Schroth therapy, home-based exercise, home program, physiotherapeutic scoliosis-specific exercises (PSSE), adherence, assistive devices, exercise system, digital tools, eHealth

## Abstract

**Background:**

Conservative scoliosis therapy in the form of assisted physiotherapeutic scoliosis exercises is supplemented by self-contained training at home, depending on the approach (eg, Schroth, the Scientific Exercises Approach to Scoliosis). Complex exercises, lack of awareness of the importance of training, and missing supervision by therapists often lead to uncertainty and reduced motivation, which in turn reduces the success of home-based therapy. Increasing digitalization in the health care sector offers opportunities to close this gap. However, research is needed to analyze the requirements and translate the potential of digital tools into concrete solution concepts.

**Objective:**

The aim of this study is to evaluate the potential for optimizing home-based scoliosis therapy in terms of motivation, assistive devices, and digital tools.

**Methods:**

In collaboration with the Institute of Physiotherapy at the Jena University Hospital, a survey was initiated to address patients with scoliosis and physical therapists. A digital questionnaire was created for each target group and distributed via physiotherapies, scoliosis forums, the Bundesverband für Skoliose Selbsthilfe e. V. newsletter via a link, and a quick response code. The survey collected data on demographics, therapy, exercise habits, motivation, assistive devices, and digital tools. Descriptive statistics were used for evaluation.

**Results:**

Of 141 survey participants, 72 (51.1%; n=62, 86.1%, female; n=10, 13.9%, male) patients with scoliosis with an average age of 40 (SD 17.08) years and 30 scoliosis therapists completed the respective questionnaires. The analysis of home-based therapy showed that patients with scoliosis exercise less per week (2 times or less; 45/72, 62.5%) than they are recommended to do by therapists (at least 3 times; 53/72, 73.6%). Patients indicated that their motivation could be increased by practicing together with friends and acquaintances (54/72, 75%), a supporting therapy device (48/72, 66.7%), or a digital profile (46/72, 63.9%). The most important assistive devices, which are comparatively rarely used in home-based therapy, included balance boards (20/72, 27.8%), wall bars (23/72, 31.9%), mirrors (36/72, 50%), and long bars (40/72, 55.6%). Therapists saw the greatest benefit of digital tools for scoliosis therapy in increasing motivation (26/30, 87%), improving home therapy (25/30, 83%), monitoring therapy progress (25/30, 83%), and demonstrating exercise instructions (24/30, 80%).

**Conclusions:**

In this study, we investigated whether there is any potential for improvement in home-based scoliosis therapy. For this purpose, using online questionnaires, we asked patients with scoliosis and therapists questions about the following topics: exercise habits, outpatient and home-based therapy, motivation, supportive devices, and digital tools. The results showed that a lack of motivation, suitable training equipment, and tools for self-control leads to a low training workload. From the perspective of the patients surveyed, this problem can be addressed through community training with friends or acquaintances, a supportive therapy device, and digital elements, such as apps, with training instructions and user profiles.

## Introduction

### Background

The term “scoliosis” is used to describe a structural 3D deformation of the spine with lateral deviations and rotations. Its severity is classified by the Cobb angle (degree of curvature) [1]. In Germany, more than 900,000 people are affected by scoliosis [2]. It is the most common spinal disease in children and adolescents [3], with growth spurts being high-risk phases for the development or worsening of scoliosis [4]. Regarding sex distribution, there is a clear tendency toward the female sex when they have a Cobb angle of 20° requiring treatment. This tendency increases with an increasing Cobb angle. In various studies, ratios (female to male) between 1.5:1 and 11.6:1 have been determined [3,5,6]. In terms of age groups, scoliosis is divided into 4 groups, infantile (1-3 years), juvenile (4-10 years), adolescent (11-18 years), and adult (over 18 years) [3], with adolescent expression being the most common form worldwide with a prevalence of 0.47-5.2 [7]. Depending on the severity of the curvature, symptoms such as back pain [8]; changes in posture in the form of shoulder, chest, and pelvic asymmetries [4]; deformations of the rib cage; and, in the case of pronounced curvatures, restrictions in heart and lung function may occur [4,8-10].

The therapeutic approach depends on the patient’s age and the extent of the deformity. Mild scoliosis (Cobb angle up to 20°) does not require therapeutic measures in most cases, except for education and motivation to be physically active. Moderate scoliosis (Cobb angle 20°-40°) is treated conservatively with scoliosis-specific braces and physiotherapeutic scoliosis-specific exercises (PSSE). In the case of severe scoliosis (Cobb angle of 40° or more), surgical interventions are used depending on the localization of the scoliosis and the patient’s age [10-12]. The most important approach in which patients with scoliosis can actively and independently participate in therapy is PSSE. The International Society on Scoliosis Orthopaedic and Rehabilitation Treatment (SOSORT) guidelines recommend PSSE in the form of outpatient physical therapy or 3- to 6-week scoliosis intensive rehabilitation (SIR) programs in specific facilities, depending on the Cobb angle [12,13]. The core elements of the therapy should be 3D autocorrection, training in activities of daily living (posture while sitting, standing, walking), stabilization of the corrected posture, and education of the patient [12]. Within the past few decades, various approaches have been developed on this basis, of which Schroth therapy, the Scientific Exercises Approach to Scoliosis (SEAS), side-shift therapy, Lyon, Dobosiewicz’s method (DoboMed), Functional Independent Treatment for Scoliosis (FITS), and the Barcelona Scoliosis Physical Therapy School (BSPTS) are among the most important. For almost all these forms of therapy, complementary, independent, and permanent home-based training can be used [14]. The positive effects of self-contained regular training sessions at home have been proven in various studies [15-18]. Nevertheless, compared to training sessions assisted by therapists, some of the results were worse [19,20]. Particularly critical factors in this context may be patient adherence and inaccurately performed exercises in an unsupervised environment [21]. Especially in home-based training, adherence is significantly influenced by motivation, belief in the benefits of exercise, a lack of monitoring, and complexity of exercises [22,23]. Increasing digitization in the health care sector offers opportunities to address some of these issues.

### Study Aims

The aim of this study is to identify the potential for optimizing home-based scoliosis therapy in terms of motivation, assistive devices, and digital tools. To represent the initial situation as holistically as possible, questionnaires were created for both patients with scoliosis (PQ) and scoliosis therapists (TQ). A survey of these target groups in Germany was intended to answer the following 4 core questions:

- How many training sessions are recommended for patients with scoliosis at home (PQ and TQ), and how often do they really exercise (PQ)?
- How motivated are patients with scoliosis to exercise at home (PQ and TQ)? Can their motivation be increased, and if so, how (PQ)?
- Which training devices are primarily used at home (PQ), and which are rated as helpful (PQ and TQ)?
- Is there interest in digital assistance, and if so, which functions would have to be realized (PQ and TQ)?

## Methods

### Survey Design

In collaboration with the Institute of Physiotherapy at the Jena University Hospital, 2 standardized online questionnaires were created using the LimeSurvey tool in order to survey scoliosis therapists (36 questions) and patients with scoliosis (33 questions) in Germany. All questions were coded for evaluation with regard to the target group surveyed (patient or therapist) and the respective topic (eg, PA01, which means PQ, topic 1 [A], and question 1, and TC02, which means TQ, topic 3 [C], and question 2; see Multimedia Appendices 1 and 2). The TQ consisted of 16 closed-ended, 14 semi-open-ended, and 6 open-ended questions and comprised 7 topics: patient groups and therapy methods, outpatient therapy, home-based therapy, communication, assistive devices, digital tools, and general questions. The PQ consisted of 21 closed-ended, 5 semi-open-ended, and 8 open-ended questions and comprised 8 topics: general questions about scoliosis, exercise habits, motivation, communication, assistive devices, digital tools, dealing with scoliosis, and general data. When developing the questionnaires, care was taken to keep them as short and simple as possible in order to achieve a high response rate and to make it easier for younger respondents in particular to answer the questions. The structure of the questionnaires had an increasing thematic depth within the survey and within a topic. Furthermore, decision questions were omitted in order to inquire about the personal attitude of the probands to the topics. Five-point Likert scales (19/69, 27.5%, of all questions) with verbally coded response options were implemented for the study of personal attitudes. An odd number of items were chosen so as not to force a decision. Furthermore, partial nonresponse answers were allowed when dealing with topics that could not be answered definitively (eg, evaluation of a form of therapy that the respondent does not know). This was intended to allow extensive content to be evaluated in the shortest time possible. In addition, many questions were linked to personal experiences in order to enable participants to quickly access the thematic focal points. As a time guideline, 10 minutes were provided for the PQ and 15 minutes for the TQ.

### Ethical Considerations

This study was reviewed by the data protection officers of the Ethics Committee at the Medical Faculty of Leipzig University and found to be of no concern. Since only anonymized data sets were provided and no re-identification was performed by the users of the data sets, there was no obligation to refer the study to an ethics committee formed according to Saxon state law. On the home page of the respective questionnaire, the topic and objective of the study were presented and the research institution conducting the study was named. The participants were informed that this was a research project and that the survey would be conducted anonymously. Before starting, all participants had to agree to the privacy policy, which was integrated via a macro and provided information about data evaluation, data subject rights, and contact persons, among other things.

### Recruitment

The distribution of the questionnaires in the patient and therapist environments was carried out in cooperation with the *Bundesverband für Skoliose Selbsthilfe e. V.* and the Physiotherapeutic Institute of the Jena University Hospital. To reach as broad a spectrum of subjects as possible, the questionnaires were distributed via scoliosis forums, direct contact, flyers with quick response (QR) codes for display in therapeutic facilities, and via the *Bundesverband für Skoliose Selbsthilfe e. V.* newsletter during the period from October 27, 2020, to June 30, 2021.

### Statistical Analysis

Data were analyzed based on descriptive statistics. For this purpose, on the one hand, frequency distributions were created, and on the other hand, the Likert scale–coded questions were evaluated using the following approach: The individual item responses of the 5-point scales were assigned point values (from 0=“not motivating at all” to 5=“very motivating”), and based on this, a sum score was calculated for the overall scale. Subsequently, the percentage of the calculated points (sum score) out of the maximum-possible points was determined. To indicate rejection or agreement as a percentage, some of the items were divided into disagreement items (eg, “not motivating at all” and “rather not motivating”) and agreement items (eg, “rather motivating” and “very motivating”), and then their proportion of the total was calculated. All free-text responses were evaluated individually and analyzed with respect to co-occurrence. Depending on the question, the patients with scoliosis were also divided into 5 age categories, inspired by the scoliosis-specific age distribution: 1-10 years (children), 11-18 years (adolescents), 19-30 years (young adults), 31-50 years, and over 50 years. Due to the low participation of those under 11 years of age, the infantile and juvenile groups were combined, while the group of people over 18 years (adults) was further divided due to the large number of participants.

## Results

### Response

The survey was based on 2 questionnaires with a total of 141 participants. The PQ was filled out by a total of 97 (68.8%) participants, 72 (74.2%) of whom answered all questions. The TQ was filled out by a total of 44 (31.2%) persons, 30 (68.2%) of whom answered all questions. All incomplete questionnaires were excluded from the analysis, so overall, 102 (72.3%) fully completed surveys were analyzed in this study.

### Demographics, Health Status, and Therapy

#### Patients

Of the 72 patients with scoliosis, 62 (86.1%) were female and 10 (13.9%) were male. The average age of the respondents was 40 (SD 17.08) years [PH01]. Broken down by age group, the distribution was as follows: up to 10 years (1/72, 1.4%), 11-18 years (9/72, 12.5%), 19-30 years (14/72, 19.4%), 31-50 years (22/72, 30.6%), and over 50 years (26/72, 36.1%). People between the ages of 7 and 79 years participated [PA02]. Regarding the Cobb angle, patients with scoliosis from all ranges were represented in our study, with Cobb angles above 50° being the most common (17/72, 23.6%), followed by 11°-20° (11/72, 15.3%). In addition, 12 (16.7%) patients responded with “I don’t know” [PA06]. In addition, of the 72 patients with scoliosis, 12 (16.7%) had already undergone surgery for their scoliosis [PA07] and 18 (25%) wore a brace [PA05].

The majority of patients with scoliosis were in therapeutic treatment for more than 2 years (56/72, 77.8%) [PA03] and attended scoliosis therapy once a week or less (61/72, 84.7%) [PB01]. On average, most patients with scoliosis exercised for up to 45 minutes in 1 physiotherapy session (60/72, 83.3%) [PB04] and up to 30 minutes in 1 home-based session (55/72, 76.4%) [PB05]. The most frequently used therapeutic approach in physiotherapy or at home was Schroth therapy (63/72, 87.5%), followed by spiral dynamics (17/72, 23.6%). The BSPTS, DoboMed, SEAS, FITS, and side-shift therapy were not known to more than 97% (70/72) of patients with scoliosis [PB07]. Other therapy methods mentioned with a maximum of 3 votes each (3/72, ≤4.2%) were yoga, fascial training, Vojta therapy, *Klappsches Kriechen*, Bobath therapy, osteopathy, sling table, manual therapy, fitness training, swimming, climbing, chiropractic, medical training therapy (MTT), proprioceptive neuromuscular facilitation (PNF), *Rota* therapy, Dorn therapy, massage, *fango* therapy, and acupuncture [PB10].

#### Therapists

The survey of the 30 scoliosis therapists showed that the most common age group of patients with scoliosis in their practices is 10-14 years (25/30, 83.3%), followed by 15-18 years (19/30, 63.3%) and over 50 years (9/30, 30%) [TA01]. The most frequently used therapy methods were Schroth therapy (29/30, 96.7%) and spiral dynamics (6/30, 20%). The following were also mentioned, each with a maximum of 2 votes (2/30, ≤6.7%): stabilization exercises, Vojta therapy, functional training, manual therapy, cupping, functional patterns by Naudi Aguilar, fascia therapy, applied kinesiology, therapeutic climbing, osteopathy, gyrotonic expansion system, yoga, and the Hancke concept [TA04]. The majority of the therapists’ patient base had been in treatment for at least 1 year (16/30, 53.3%) [TB01] and had been in practice on average once a week or more (25/30, 83.3%) [TB02]. A guided training session lasted between 16 and 30 minutes for most therapists (19/30, 63.3%) [TB04].

### Home-Based Therapy

**Figure 1:**
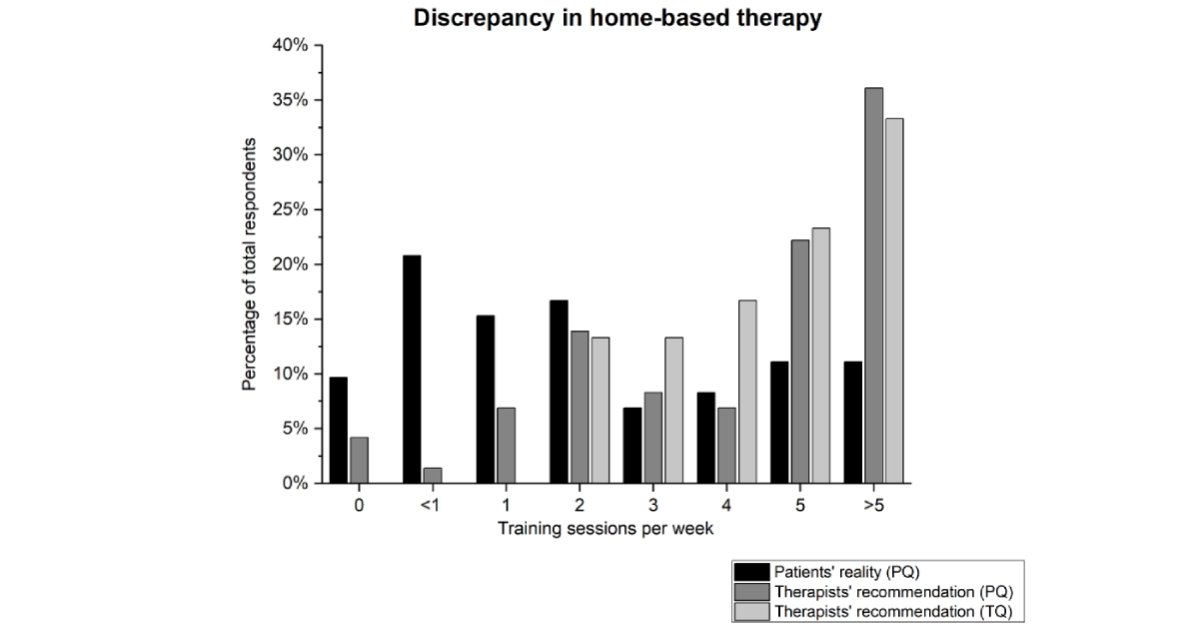
Comparison of weekly training sessions performed by patients with scoliosis at home and recommendations of therapists in this regard. To create the figure, the results of 2 questions from PQ (“How often do you do additional therapy exercises at home for your scoliosis?” [PB02; black] and “How often did your therapist recommend you to do exercises at home?” [PB03; dark gray]) and 1 question from TQ (“How often do you usually recommend additional home exercise sessions to your patients for physical therapy?” [TC01; light gray]) were used. PB02: PQ, topic 2, question 2; PB03: PQ, topic 2, question 3; PQ: questionnaire for patients with scoliosis; TC01: TQ, topic 3, question 1; TQ: questionnaire for scoliosis therapists.

In the case of scoliosis home training, there was an opposite trend: Although the majority of patients with scoliosis trained twice or less per week (45/72, 62.5%) [PB02], the majority of therapists recommended at least 3 training sessions per week (PQ: 53/72, 73.6%; TQ: 26/30, 86.7%) [PB03, TC01]; see Figure 1.

For further substantiation, the interviewed therapists were asked to rate the dependence of their recommendations on 4 parameters using a 5-point Likert scale: (1) Cobb angle, (2) age, (3) personal motivation, and (4) cognitive aptitude. The survey of the 30 therapists showed that personal motivation (26/30, 85.3%) and cognitive aptitude (25/30, 82%) were the most important factors from our selection [TC02].

### Motivation

**Figure 2:**
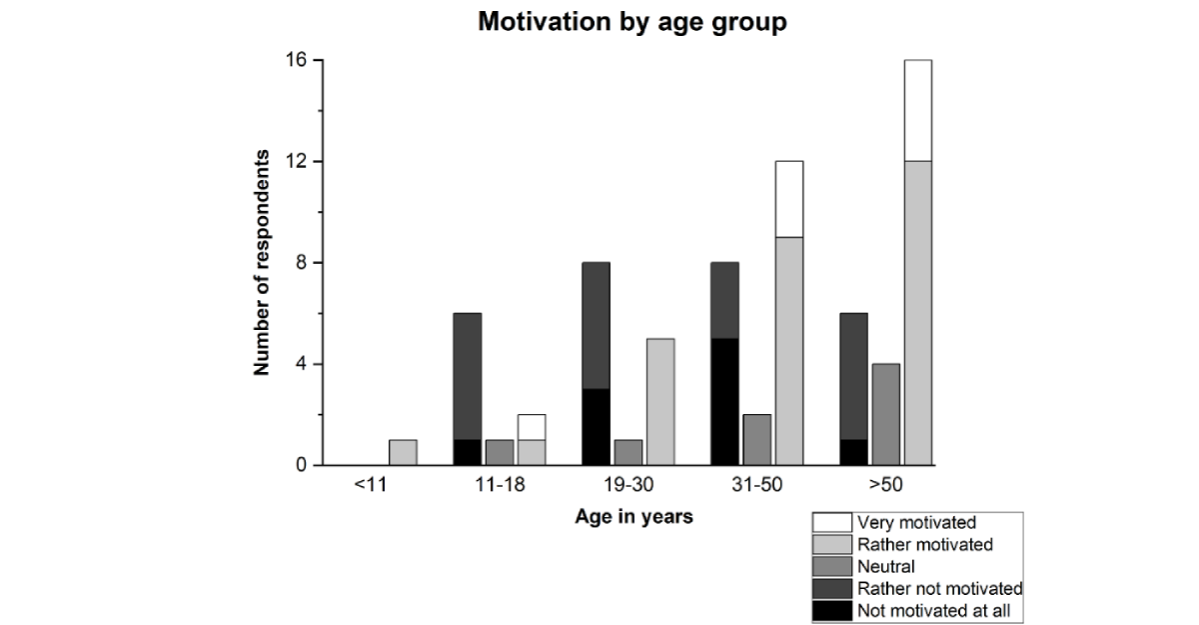
How motivated are patients with scoliosis to perform their exercises, broken down by age group? The figure is based on the results of 1 question from PQ: “How motivated are you in general to do your exercises?” [PC01]. The disagreement items (“not motivated at all” and “rather not motivated”) are visualized in black and dark gray, respectively, while the agreement items (“rather motivated” and “very motivated”) are visualized in light gray and white, respectively, each stacked. PC01: PQ, topic 3, question 1; PQ: questionnaire for patients with scoliosis.

We asked how motivated patients with scoliosis were in general to perform their exercises (see Figure 2). Analysis of the data showed that children, adolescents, and young adults in particular are less motivated. This trend reversed with increasing age in our survey. According to their own statements, people aged 50 years and above had the greatest motivation [PC01].

A similar relationship emerged in the therapist survey. According to the therapists questioned, children and adolescents were the least motivated to perform home-based therapy [TB06].

**Figure 3:**
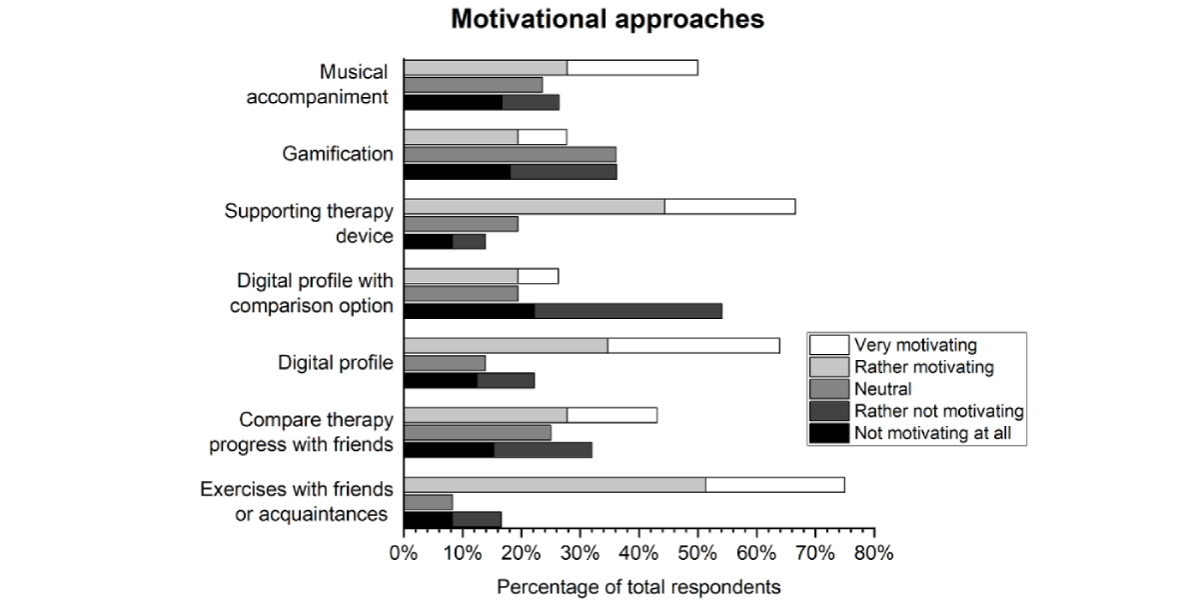
What would motivate patients with scoliosis to perform their exercises? The content of the graph is based on the results of voting from the PQ: “Please indicate how motivating you would find the following features for your scoliosis exercises” [PC02]. Here, the disagreement items (“not motivating at all” and “rather not motivating”) are visualized in black and dark gray, respectively, while the agreement items (“rather motivating” and “very motivating”) are visualized in light gray and white, respectively, each stacked. PC02: PQ, topic 3, question 2; PQ: questionnaire for patients with scoliosis.

In a second question on motivation, patients with scoliosis were asked to rate a preselection of features in terms of their motivational potential using a 5-point Likert scale. The most popular features (agreement items only) for increasing motivation were “exercises with friends or acquaintances” (54/72, 75%), “supporting therapy device” (48/72, 66.7%), and “digital profile” (46/72, 63.9%). The worst score was for “digital profile with comparison option” (19/72, 26.4%). The greatest uncertainty was seen in “gamification” (“neutral,” or “neither motivating nor not motivating”; 26/72, 36.1%) [PC02]; see Figure 3.

The top 4 features in the 3 age groups of up to 30 years (least motivated) were “exercises with friends or acquaintances” (18/24, 75%), “supporting therapy device” (14/24, 58.3%), “digital profile” (13/24, 54.2%), and “musical accompaniment” (13/24, 54.2%) [PC01].

### Assistive Devices

In home-based training, “cushions and gymnastic mats” (58/72, 80.6%), “stools and chairs” (50/72, 69.4%), and “sand and rice bags” (48/72, 66.7%) were used most frequently. “Tables,” in contrast, were used by just a quarter of respondents (18/72, 25%) [PE01].

**Figure 4:**
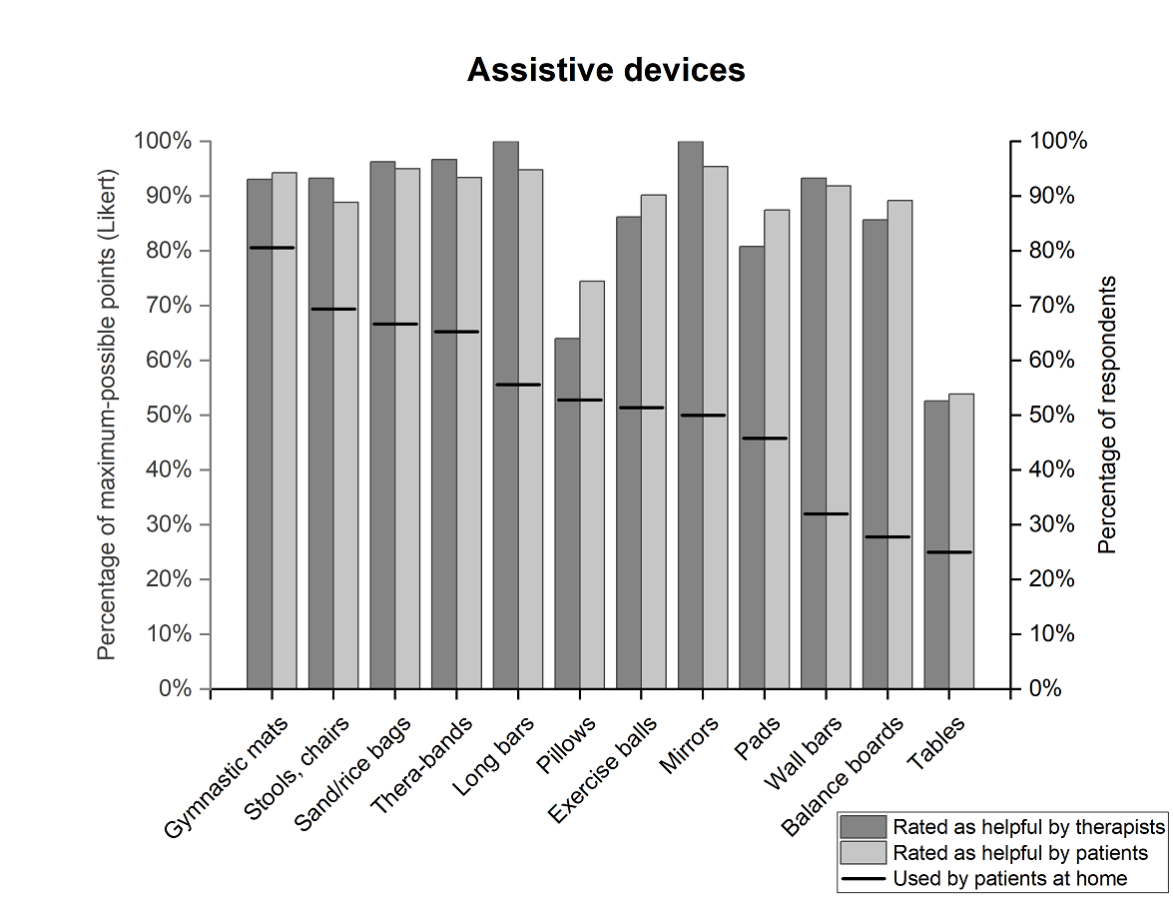
Which assistive devices do patients with scoliosis use for training at home, and which do patients and therapists rate as helpful for scoliosis therapy? For the creation of the figure, the results of 2 questions from PQ (“Where do you use, or where would you like to use, which of the predefined tools?” [PE01; black lines] and “How helpful do you find the mentioned devices?” [PE02; light gray]) and 1 voting from TQ (“Please rate how helpful the mentioned assistive devices are for scoliosis therapy” [TE03; gray]) were used. The Likert distribution was calculated excluding the answer “I do not use.” PE01: PQ, topic 5, question 1; PE02: PQ, topic 5, question 2; PQ: questionnaire for patients with scoliosis; TE03: TQ, topic 5, question 3; TQ: questionnaire for scoliosis therapists.

The 3 most helpful assistive devices for patients with scoliosis were “mirrors” (93.8%), “sand and rice bags” (93.3%), and “wall bars” (93.2%) [PE02]. Note that these percentages refer to the results of the Likert scale, in which scores for the answer “I do not use” were eliminated. A similar picture was shown by the therapists, who rated “mirrors” (98.7%), “sand and rice bags” (94.1%), and “long bars” (92.7%) as most helpful [TE03]. Highly valued (at least 80%) but relatively underused in home-based therapy were “wall bars,” “balance boards,” “mirrors,” “pads” (eg, foam rollers), and “long bars” [PE01, PE02, TE03]; see Figure 4.

### Digital Assistance

**Figure 5:**
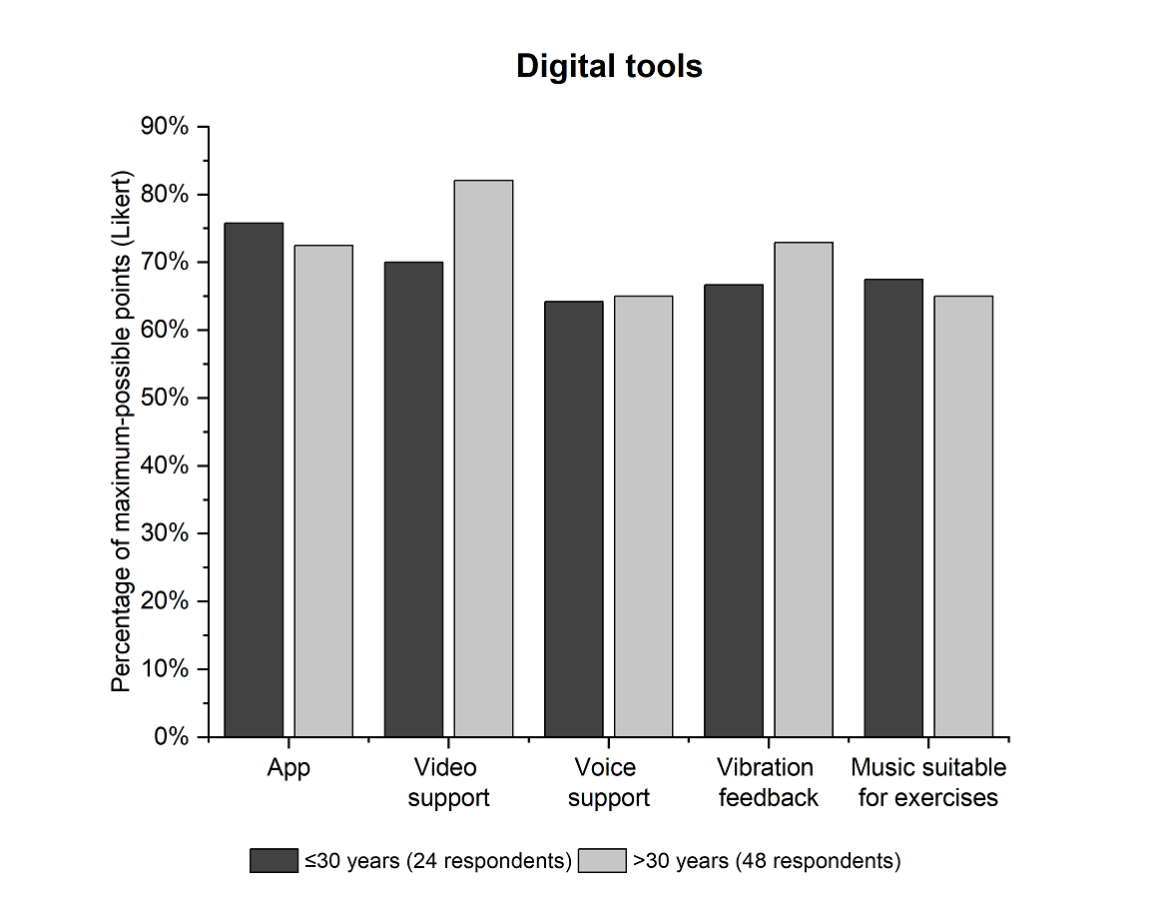
Evaluation of digital tools with regard to their usefulness in supporting scoliosis therapy. The results of the question “How helpful do you find, or would you find, the following digital tools in your exercises?” [PF02] from PQ were evaluated for the creation of the graph. The evaluation was carried out using a 5-point Likert scale, divided into 2 age groups. PF02: PQ, topic 6, question 2; PQ: questionnaire for patients with scoliosis.

Respondents were asked to rate 5 digital tools in terms of their usefulness on a 5-point Likert scale. In patients in the age groups of up to 30 years (24/72, 33.3%, respondents), the digital tools “smartphone or tablet app” (eg, exercise guide; 18/24, 75.8%), “video support” (eg, instructional video; 17/24, 70%), and “music suitable for exercises” (16/24, 67.5%) were the most popular. In patients aged 31 years or above (48/72, 66.7%, respondents), the most popular tools were “video support” (eg, instructional video; 38/48, 82.1%), “vibration feedback” (vibration when exercises are performed correctly or incorrectly; 35/48, 72.9%), and “smartphone or tablet app” (eg, exercise instructions; 34/48, 72.5%) [PF02]; see Figure 5.

The survey of therapists also revealed that digital tools, such as smartphones (18/30, 60%), watches (9/30, 30%), and tablets (7/30, 23.3%) were already used for scoliosis therapy [TF01]. The most important apps currently included “documentation of therapy progress” (18/30, 60%), “exercise instructions” (16/30, 53.3%), and “communication with the patient” (11/30, 36.7%) [TF02]. Therapists saw the greatest potential in the use of digital tools for “increasing motivation” (26/30, 87%), “improving home therapy” (25/30, 83%), “monitoring therapy progress” (25/30, 83%), and “exercise instructions” (24/30, 80%). The least convincing were “virtual therapy sessions” (15/30, 50%) [TF03].

Based on the survey on the potential of digital tools, therapists were also asked to evaluate necessary parameters for improvement of home-based therapy. The tracking of “position and movement of certain body parts” (27/30, 90%) was seen as the most important parameter, followed by the measurement of “vital capacity” (13/30, 43.3%) [TF05]. Therapists also preferred the following variants for a therapy-supporting exchange with patients: “exercise instructions as videos” (26/30, 86.7%), “exercise recordings as videos” (24/30, 80%), “sensor data on position and movement” (16/30, 53.3%), and “exercise instructions as pictures” (14/30, 46.7%) [TF07].

## Discussion

### Principal Findings

The aim of this study was to evaluate the potential for optimizing home-based scoliosis therapy in terms of motivation, assistive devices, and digital tools. For this purpose, the topics of training habits, motivation, assistive devices, and digital assistance were addressed in online questionnaires. To gain the most comprehensive insight possible, both patients with scoliosis and scoliosis therapists were surveyed.

In line with the literature, the percentage of female respondents in our patient survey was much higher, with a ratio of 6.2:1 [3,5,6]. Additionally, both questionnaires revealed that Schroth therapy is the most widespread and popular therapy method in Germany. Its effectiveness as part of conservative scoliosis therapy has been proven in numerous publications [24-26].

In addition to physiotherapeutic treatments, the commitment of patients to deal with scoliosis and to exercise regularly on their own at home is decisive for the success of conservative scoliosis therapy [27]. Depending on age and the Cobb angle, patients with scoliosis are entitled to various types of treatment according to the *Heilmittel-Richtlinie* in Germany, the costs of which are covered by health insurance [28]. These are primarily physiotherapeutic approaches, such as manual therapy, since there are no separate remedy positions for scoliosis therapies [29]. These therapies cover some of the necessary training, yet therapists additionally recommend a continuous and comprehensive home exercise program. Their recommendations depend on the motivation and cognitive aptitude of their patients. Based on this initial situation, we compared recommendations and the reality for home-based training. A clear trend emerged, which is almost indirectly proportional: patients with scoliosis exercise significantly less at home than therapists recommend. Reasons for this opposite trend may include a lack of time and motivation [23,30], the complexity or number of exercises, forgetting training sessions [23], uncertainty in performing exercises, fear of aggravation, and pain [31].

The survey of therapists showed that children, adolescents, and young adults, especially, undergo physiotherapy treatment for their scoliosis. Because growth is not yet complete, the chances of success of therapy are the highest in this age group [32,33]. However, this is countered by the fact that it is precisely in these age groups that motivation for scoliosis-specific exercises appears to be the lowest, both from the perspective of the patients with scoliosis surveyed and from that of the therapists. This trend may occur because young patients prioritize other aspects in everyday life, which is also reflected in the participation in our survey. An appropriate way to educate children, adolescents, and young adults about age-related problems due to scoliosis has not yet been found. Furthermore, scoliosis-related pain in these age groups is still too low to raise awareness of the importance of training. The increase in pain with increasing age [34] could be a reason for the greater motivation of older-age groups. According to the patients with scoliosis surveyed, motivation could be increased by joint training sessions with friends and acquaintances, a specific therapy device, and a digital profile. In this context, it should be noted that although the digital profile received the most votes for “very motivating,” it only ranked third overall. Half of the respondents still found musical accompaniment motivating. Although community training and musical accompaniment can be partially implemented on their own, new approaches are needed for a specific therapy device and digital profile. The comparison with other patients was not felt to be motivating. Although this can spur one on, it can also be discouraging if one either cannot keep up or one lacks “digital friends” [35]. In addition, a meaningful comparison is difficult to realize due to the high degree of individualization of therapy. The greatest uncertainty was found in the “combining the exercises with a game” approach. The reason for this could be that this approach was seen without a digital reference (gamification) and that the patients with scoliosis surveyed could not imagine combining their current therapy with a game. The question intended to obtain insights into the participants’ opinion on the transfer of training content into a digital environment (eg, an app) with playful elements or visualizations. An increase in motivation can be achieved through the fun of the game as well as through high scores and digital reward systems (eg, badges, points) when completing tasks. The average age (40 years) of the respondents is unlikely to have influenced the answer in this respect, as 44% of people who occasionally or regularly play video games in Germany are aged 40 years or above. The situation is similar with regard to sex and gender, as the ratio between male and female video gamers in Germany is relatively balanced: around 48% are female and 52% are male [36]. Several studies in the past few years have shown that gamification approaches can have a motivating effect in rehabilitation [37]. The literature identifies personal analyses to progress, data tracking, a competitive environment [35,37], and a sense of community, autonomy, and competence [38] as crucial factors for motivation. Wibmer et al [39] explicitly investigated the potential of gamification in scoliosis therapy. They were able to show that it is possible to increase motivation and precision when performing scoliosis-specific exercises. However, this effect depends on how varied and adaptable the games are designed and thus can also quickly become invalid [39]. A successful gamification approach requires that patients be involved in the development of the game from the beginning and that the possibility of cheating within the game be excluded. Furthermore, different game environments appeal to different groups of people. This should be considered during development [35].

Another influencing factor for the optimization of home-based therapy could be assistive devices that can be used for training. Langensiepen et al [15] reported that the use of side-alternating vibration plates can lead to an improvement in home-based training. Our survey showed that patients with scoliosis mainly use gymnastic mats and bands, sand and rice bags, stools, and chairs at home. These tools are inexpensive, are easy to obtain, and require little storage space. However, patients with scoliosis and therapists found mirrors to be the most helpful of our selection of tools. This offers the advantage of self-control when performing exercises, which is especially important at home [40]. Nevertheless, mirrors were used by only half of the patients with scoliosis we interviewed. One reason for this could be that there is a lack of suitable installation possibilities in private households or that there is not enough space in front of the existing mirrors to perform the exercises. The same applies to wall bars, balance boards, pads, and long poles, which are popular with both patients with scoliosis and therapists but are used relatively little at home. Overall, both groups found 10 (more than 80% approval) of our 12 mentioned tools useful for scoliosis therapy. However, only 1 in 12 devices was used by at least 80% of patients with scoliosis at home. A supportive therapy device that meets the requirements of home training and, if necessary, combines several training options of the aforementioned devices could thus contribute to improving scoliosis therapy. However, it is important that the therapy device not increase the complexity of the training.

After examining motivation and aids, we looked at the potential of digital tools in the last section. Currently, multisensory, smartphone-based systems for improving adherence [41], pressure sensor systems for adapted corsets [42], and apps for Cobb angle measurement [43-45] and therapy support [46] are used in scoliosis therapy. These can be used advantageously for rehabilitation, especially in the areas of visualization [47], networking, information exchange, monitoring [48], and motivation increase [49]. Based on this, we asked patients with scoliosis and therapists which digital tools they thought would be helpful for scoliosis therapy. Our preselection of 5 tools revealed different preferences, depending on the age group. Although a suitable smartphone or tablet app (eg, with exercise instructions) was most preferred by patients in the age groups of up to 30 years, those over 30 years old would particularly like video support (eg, in the form of instructional videos). Overall, the response was predominantly positive for all tools that serve to support correct exercise execution. Training can lead to incorrect loads or incorrect execution, particularly at home without the presence of a therapist, which can have a negative effect on therapy. In addition to mirrors, which patients with scoliosis can use during therapy, there is a lack of opportunities for self-monitoring at home. In our questionnaire on home-based therapy, the therapists therefore stated that the tracking of positions and movements of the body is a priority. They also saw great potential in increasing motivation, monitoring therapy progress, and optimizing exercise instructions through digital tools. Due to the COVID-19 pandemic, we also sought opinions on virtual therapy sessions. This approach was considered useful by only half of the therapists.

### Limitations

Our survey consisted of online questionnaires that were distributed primarily via digital media (forums, social media, QR codes, etc). It can therefore be assumed that the survey was primarily completed by technically skilled respondents. Some of the patients with scoliosis and therapists may have been excluded. Nevertheless, this methodology allowed a larger sample to be reached. Another limitation of the online questionnaires is that answers may have been given that were not true or that people who neither have scoliosis nor treat it participated. The small sample size of the survey was due to the available boundary conditions. Since the survey was conducted within the framework of a 2-year research project, the capacity for the acquisition of participants and the period for data collection were limited. The goal was to integrate the results into the development process of the research project. Another potential limitation of this study is that the average age of our patient survey was 40 years. Children, adolescents, and young adults were thus comparatively underrepresented, which is why a downstream study with an adapted design that focuses exclusively on this target group is conceivable. In addition, it is possible that the youngest participants in our survey completed the questionnaires together with their parents. In this case, the answers may have been influenced by the parents. Another limitation is the fact that the study was limited to Germany. This raises the possibility that patients with scoliosis and therapists in other countries might have given different answers to the questionnaires, depending on the health care system or local therapy methods. Furthermore, the fact that significantly fewer therapists than patients with scoliosis participated in our survey had a limiting effect on the study. However, it must be considered that there are also significantly more people with scoliosis in Germany than therapists treating them.

### Conclusion

In this study, we investigated whether there is any potential for improvement in home-based scoliosis therapy. For this purpose, via online questionnaires, we asked patients with scoliosis and therapists questions about the following topics: exercise habits, outpatient and home-based therapy, motivation, supportive devices, and digital tools. The results showed that a lack of motivation, suitable training equipment, and tools for self-control leads to a low training workload. From the perspective of the patients with scoliosis surveyed, this problem can be addressed by community training with friends or acquaintances, a supportive therapy device, and digital elements, such as apps, with training instructions and user profiles.

## Appendix

Multimedia Appendix 1 Patient questionnaire.

Multimedia Appendix 2 Therapist questionnaire.

## Abbreviations

BSPTS: Barcelona Scoliosis Physical Therapy School
DoboMed: Dobosiewicz’s method
FITS: Functional Independent Treatment for Scoliosis
PQ: questionnaire for patients with scoliosis
PSSE: physiotherapeutic scoliosis-specific exercises
QR: quick response
TQ: questionnaire for scoliosis therapists
