# Supplementary material for: Improving Home-Based Scoliosis Therapy: Findings From a Web-Based Survey
Source: JMIR Rehabil Assist Technol. 2023 Aug 4;10:e46217. doi: 10.2196/46217 (PMC10439467; doi:10.2196/46217)
Supplement: Multimedia Appendix 1 [file rehab_v10i1e46217_app1.pdf]

Nutzerbefragung zum Therapieverhalten von Skoliose-Patienten

Im Rahmen eines Forschungsprojektes erforscht das Fraunhofer Institut für Werkzeugmaschinen und Umformtechnik (IWU) in Dresden aktuell die Entwicklung eines Heimtherapie Ansatzes für Skoliose-Patienten. Dazu werden zunächst Nutzerbefragungen bei Skoliose-Patienten durchgeführt, die in therapeutischer Behandlung sind oder waren.

Ziel dieses Fragebogens ist es Einblicke in das aktuelle Therapieverhalten von Patienten in Bezug auf Motivation zur Therapie, den verwendeten Hilfsmitteln, der Einstellung gegenüber digitalen Hilfsmitteln und der Kommunikation mit dem Therapeuten oder der Therapeutin zu bekommen.

Wenn Sie sich nicht mehr in Skoliosebehandlung befinden, versetzen Sie sich bitte in die Zeit zurück und beantworten die Fragen so, als würden Sie Ihre Skoliose aktuell behandeln.

Die in diesem Fragebogen erhobenen Daten sind nicht personenbezogen und die Auswertung aller gegebenen Antworten erfolgt anonym. Wir bedanken uns herzlich für Ihre wertvolle Zeit und Ihren wichtigen Beitrag zu diesem Thema.

Die Beantwortung des Fragebogens dauert maximal 10 Minuten.

In dieser Umfrage sind 33 Fragen enthalten.

Allgemeine Fragen zu Ihrer Skoliose

In diesem Teil wollen wir erst einmal ein paar allgemeine Dinge über Ihre Skoliose erfahen. Bitte beantworten Sie die Fragen so, als würden Sie sich momentan in Skoliosebehandlung befinden.

PA01

In welchem Alter haben Sie mit der Skoliosebehandlung begonnen? *

Bitte geben Sie Ihre Antwort hier ein:

PA02

Wie alt sind Sie jetzt? *

Bitte geben Sie Ihre Antwort hier ein:

PA03

Wie lang sind Sie schon bei der Skoliosebehandlung oder wie lang waren Sie es? *

Bitte wählen Sie nur eine der folgenden Antworten aus:

- ◯ Weniger als 2 Wochen
- ◯ 2 bis 5 Wochen
- ◯ 6 - 12 Wochen
- ◯ 3 - 6 Monate
- ◯ 7 - 12 Monate
- ◯ Länger als 1 Jahr
- ◯ Länger als 2 Jahre

PA04

Wie groß war Ihr Cobb-Winkel vor der Therapie? *

Bitte wählen Sie nur eine der folgenden Antworten aus:

- ◯ 0 - 10°
- ◯ 11 - 20°
- ◯ 21 - 30°
- ◯ 31 - 40°
- ◯ 41 - 50°
- ◯ Über 50°
- ◯ Über 80°
- ◯ weiß ich nicht

Der Cobb-Winkel bestimmt den Grad der Skoliose. Bitte gebe Sie Ihren Cobb-Winkel-Bereich ein, wenn Sie ihn kennen.

PA05

Tragen Sie neben der Therapie-Übungen ein Skoliose-Korsett? *

Bitte wählen Sie nur eine der folgenden Antworten aus:

- ◯ Ja, ein Ganztagskorsett
- ◯ Ja, ein Nachtkorsett
- ◯ Nein

PA06

Wie groß ist Ihr Cobb-Winkel jetzt oder wie groß war er nach der Therapie? *

Bitte wählen Sie nur eine der folgenden Antworten aus:

- ◯ 0 - 10°
- ◯ 11 - 20°
- ◯ 21 - 30°
- ◯ 31 - 40°
- ◯ 41 - 50°
- ◯ Über 50°
- ◯ Über 80°
- ◯ weiß ich nicht

Der Cobb-Winkel bestimmt den Grad der Skoliose. Bitte gebe Sie Ihren Cobb-Winkel-Bereich ein, wenn Sie ihn kennen.

PA07

Wurden Sie wegen Ihrer Skoliose schon einmal operiert? *

Bitte wählen Sie nur eine der folgenden Antworten aus:

- ◯ Ja
- ◯ Nein

Übungsgewohnheiten

In diesem Abschnitt wollen wir mehr darüber erfahren wann, wie und wo Sie Ihre Übungen machen oder gemacht haben. Bitte beantworten Sie die Fragen so, als würden Sie sich

momentan in Skoliosebehandlung befinden.

PB01

Wie oft gehen Sie zur Skoliose-Therapie? *

Bitte wählen Sie nur eine der folgenden Antworten aus:

- ◯ Gar nicht
- ◯ weniger als 1 Mal pro Wiche
- ◯ 1 Mal pro Woche
- ◯ 2 Mal pro Woche
- ◯ 3 Mal pro Woche
- ◯ 4 Mal pro Woche
- ◯ 5 Mal pro Woche
- ◯ Mehr als 5 Mal pro Woche

PB02

Wie oft machen Sie zusätzlich zu Hause Therapie-Übungen gegen Ihre Skoliose? *

Bitte wählen Sie nur eine der folgenden Antworten aus:

- ◯ Gar nicht
- ◯ Weniger als 1 Mal pro Woche
- ◯ 1 Mal pro Woche
- ◯ 2 mal pro Woche
- ◯ 3 Mal pro Woche
- ◯ 4 Mal pro Woche
- ◯ 5 Mal pro Woche
- ◯ Mehr als 5 Mal pro Woche

PB03

Wie oft hat Ihnen Ihr Therapeut oder Ihre Therapeutin empfohlen, zu Hause zu üben? *

Bitte wählen Sie nur eine der folgenden Antworten aus:

- ◯ Gar nicht
- ◯ Weniger als 1 Mal pro Woche
- ◯ 1 Mal pro Woche
- ◯ 2 Mal pro Woche
- ◯ 3 Mal pro Woche
- ◯ 4 Mal pro Woche
- ◯ 5 Mal pro Woche
- ◯ Mehr als 5 Mal pro Woche

PB04

Wie lang dauert eine Sitzung bei Ihrer Physiotherapie in der Regel? *

Bitte wählen Sie nur eine der folgenden Antworten aus:

- ◯ 0 - 15 Minuten
- ◯ 16 - 30 Minuten
- ◯ 31 - 45 Minuten
- ◯ 46 - 60 Minuten
- ◯ Mehr als 60 Minuten

PB05

Wie lang machen Sie Ihre Übungen zu Hause in der Regel? *

Bitte wählen Sie nur eine der folgenden Antworten aus:

- ◯ 0 - 15 Minuten
- ◯ 16 - 30 Minuten
- ◯ 31 - 45 Minuten
- ◯ 46 - 60 Minuten
- ◯ Mehr als 60 Minuten

PB06

Wo haben Sie Ihre Übungen gelernt? *

Bitte wählen Sie alle zutreffenden Antworten aus:

- ☐ Physiotherapie
- ☐ Internet
- ☐ Bücher
- ☐ Therapiezentrum
- ☐ Sonstiges: ____________

Mehrfachauswahl möglich

PB07

Aus welchen der genannten Methoden habe Sie schon einmal Übungen gemacht? *

Bitte wählen Sie die zutreffende Antwort für jeden Punkt aus:

	bei der Physiotherapie	beim Üben zu Hause	Physiotherapie und zu Hause	kenne ich, habe aber keine Übungen gemacht	kenne ich nicht
**Schroth**	◯	◯	◯	◯	◯
**Barcelona Scoliosis Physical Therapy School (BSPTS)**	◯	◯	◯	◯	◯
**DoboMed**	◯	◯	◯	◯	◯
**SEAS**	◯	◯	◯	◯	◯
**FITS**	◯	◯	◯	◯	◯
**Side Shift Program**	◯	◯	◯	◯	◯
**Spiraldynamik**	◯	◯	◯	◯	◯

PB08

Bitte geben Sie an, wie effektiv sie die genannten Therapiemethoden einschätzen. *

Bitte wählen Sie die zutreffende Antwort für jeden Punkt aus:

	überhaupt nicht effektiv	eher nicht effektiv	weder noch	eher effektiv	sehr effektiv	kenne ich nicht oder weiß ich nicht
**Schroth**	◯	◯	◯	◯	◯	◯
**Barcelona Scoliosis Physical Therapy School (BSPTS)**	◯	◯	◯	◯	◯	◯
**DoboMed**	◯	◯	◯	◯	◯	◯
**SEAS**	◯	◯	◯	◯	◯	◯
**FITS**	◯	◯	◯	◯	◯	◯
**Side Shift Program**	◯	◯	◯	◯	◯	◯
**Spiraldynamik**	◯	◯	◯	◯	◯	◯

PB09

Bitte geben Sie an, wie sehr folgende Aussagen auf Sie zutreffen. *

Bitte wählen Sie die zutreffende Antwort für jeden Punkt aus:

	trifft überhaupt nicht zu	trifft eher nicht zu	weder noch	trifft eher zu	trifft voll zu
**Ich merke es, wenn ich meine Übungen richtig mache.**	○	○	○	○	○
**Die Übungen sind schmerzhaft für mich.**	○	○	○	○	○

PB10

Welche Therapiemethoden kennen Sie noch?

Bitte geben Sie Ihre Antwort hier ein:

Motivation

In diesem Teil möchten wir etwas über Ihre Motivation für die Skoliose-Therapie herausfinden. Bitte beantworten Sie die Fragen so, als würden Sie sich momentan in Skoliosebehandlung befinden.

PC01

Wie motiviert sind Sie allgemein Ihre Übungen zu machen? *

Bitte wählen Sie nur eine der folgenden Antworten aus:

- ○ überhaupt nicht motiviert
- ○ eher nicht motiviert
- ○ weder noch
- ○ eher motiviert
- ○ sehr motiviert

PC02

Bitte geben Sie an, wie motivierend Sie folgende Features für Ihre Skoliose-Übungen finden würden. *

Bitte wählen Sie die zutreffende Antwort für jeden Punkt aus:

	überhaupt nicht motivierend	eher nicht motivierend	weder noch	eher motivierend	sehr motivierend
**Übungen mit Freunden oder Bekannten zusammen machen**	○	○	○	○	○
**Mich beim Üben und beim Therapiefortschritt mit Freunden oder Bekannten vergleichen**	○	○	○	○	○
**Ein digitales Profil, in dem ich meinen Erfolg sehen kann**	○	○	○	○	○
**Ein digitales Profil, in dem ich meinen Erfolg sehen und mit anderen vergleichen kann ("Facebook für Skoliose")**	○	○	○	○	○
**Skoliose-Übungen im Rhythmus zur Musik**	○	○	○	○	○
**Die Übungen mit einem Spiel verbinden (Zum Beispiel Belohnungen für richtig ausgeführte Übungen)**	○	○	○	○	○
**Ein unterstützendes Therapie-Gerät für zu Hause (wie im Fitnessstudio)**	○	○	○	○	○

Kommunikation

In diesem Teil wollen wir herausfinden, wie sie mit Ihrem Therapeuten oder Ihrer Therapeutin kommunizieren und wie Sie die Kommunikation wahrnehmen. Bitte beantworten Sie die Fragen so, als würden Sie sich momentan in Skoliosebehandlung befinden.

PD01

Über welche Kommunikationswege reden Sie mit Ihrem Therapeuten oder Ihrer Therapeutin? *

Bitte wählen Sie alle zutreffenden Antworten aus:

- ☐ bei der Therapie
- ☐ per Telefon
- ☐ per E-Mail
- ☐ per Chat (WhatsApp, Telegram, Facebook etc.)
- ☐ Sonstiges: ________

Mehrfachauswahl möglich

PD02

Wie viele Versuche brauchen Sie in der Regel, um eine bestimmte Übung richtig auszuführen? *

Bitte wählen Sie nur eine der folgenden Antworten aus:

- ○ 1
- ○ 2
- ○ 3
- ○ 4
- ○ 5
- ○ Andere ________

PD03

Bitte geben Sie an, wie gut Sie die Kommunikation mit Ihrem Therapeuten oder Ihrer Therapeutin bezüglich der folgenden Punkte einschätzen. *

Bitte wählen Sie die zutreffende Antwort für jeden Punkt aus:

	sehr schlecht	eher schlecht	weder noch	eher gut	sehr gut
**Übungsanweisungen**	○	○	○	○	○
**Informationen zu meiner Skoliose**	○	○	○	○	○
**Sinn einzelner Übungen**	○	○	○	○	○
**Mein Therapiefortschritt**	○	○	○	○	○
**Wie lang (noch) ich zur Therapie muss**	○	○	○	○	○
**allgemein**	○	○	○	○	○

Geräte als Hilfsmittel

In diesem Teil wollen wir herausfinden, welche Geräte Sie bei Ihren Übungen verwenden und wie hilfreich Sie welche davon finden. Bitte beantworten Sie die Fragen so, als würden Sie sich momentan in Skoliosebehandlung befinden.

PE01

Wo nutzen Sie welche der genannten Hilfsgeräte oder wo würden Sie sie gerne nutzen? *

Bitte wählen Sie die zutreffende Antwort für jeden Punkt aus:

	bei der Physiotherapie	beim Üben zu Hause	bei beidem	bei der Physiotherapie und würde es gerne zu Hause nutzen	zu Hause und würde es gern bei der Physiotherapie nutzen	gar nicht
Polstermatten oder Gymnastikmatten	◯	◯	◯	◯	◯	◯
Unterlagen (Schaumstoffrollen etc.)	◯	◯	◯	◯	◯	◯
Sandsäckchen oder Reissäckchen	◯	◯	◯	◯	◯	◯
Kissen	◯	◯	◯	◯	◯	◯
Sprossenwand	◯	◯	◯	◯	◯	◯
Spiegel	◯	◯	◯	◯	◯	◯
lange Stäbe	◯	◯	◯	◯	◯	◯
Hocker oder Stühle	◯	◯	◯	◯	◯	◯
Tisch	◯	◯	◯	◯	◯	◯
Gymnastikball oder Medizinball	◯	◯	◯	◯	◯	◯
Balance Board	◯	◯	◯	◯	◯	◯
Bänder (Gummibänder, Therabänder etc.)	◯	◯	◯	◯	◯	◯

PE02

Wie hilfreich finden Sie die genannten Geräte? *

Bitte wählen Sie die zutreffende Antwort für jeden Punkt aus:

	überhaupt nicht hilfreich	eher nicht hilfreich	weder noch	eher hilfreich	sehr hilfreich	nutze ich nicht
**Polstermatten oder Gymnastikmatten**	◯	◯	◯	◯	◯	◯
**Unterlagen (Schaumstoffrollen etc.)**	◯	◯	◯	◯	◯	◯
**Sandsäckchen oder Reissäckchen**	◯	◯	◯	◯	◯	◯
**Kissen**	◯	◯	◯	◯	◯	◯
**Sprossenwand**	◯	◯	◯	◯	◯	◯
**Spiegel**	◯	◯	◯	◯	◯	◯
**lange Stäbe**	◯	◯	◯	◯	◯	◯
**Hocker oder Stühle**	◯	◯	◯	◯	◯	◯
**Tisch**	◯	◯	◯	◯	◯	◯
**Gymnastikball oder Medizinball**	◯	◯	◯	◯	◯	◯
**Balance Board**	◯	◯	◯	◯	◯	◯
**Bänder (Gummibänder, Therabänder etc.)**	◯	◯	◯	◯	◯	◯

PE03

Welche Geräte würden Sie sich noch als Hilfsmittel wünschen?

Bitte geben Sie Ihre Antwort hier ein:

[ ]

Digitale Hilfsmittel

In diesem Teil wollen wir herausfinden, wie Sie zum Einsatz von digitalen Tools in der Skoliose-Therapie stehen oder welche Sie vielleicht bereits nutzen. Bitte beantworten Sie die Fragen so, als würden Sie sich momentan in Skoliose Behandlung befinden.

PF01

Welche digitale Tools nutzen Sie bereits bei der Physiotherapie oder zu Hause? *

Bitte wählen Sie alle zutreffenden Antworten aus:

- [ ] Smartphone
- [ ] Tablet
- [ ] Kamera
- [ ] Uhr (digital oder auch analog)
- [ ] keine
- [ ] Sonstiges: [ ]

PF02

Wie hilfreich finden oder fänden Sie die folgenden digitalen Tools bei Ihren Übungen? *

Bitte wählen Sie die zutreffende Antwort für jeden Punkt aus:

	überhaupt nicht hilfreich	eher nicht hilfreich	weder noch	eher hilfreich	sehr hilfreich
**Smartphone- oder Tablet App (z.B. Übungsanleitung)**	○	○	○	○	○
**Sprachunterstützung (z.B. Stimme die mir sagt, wenn ich eine Übung falsch mache)**	○	○	○	○	○
**Videounterstützung (z.B. Anleitungsvideo)**	○	○	○	○	○
**Vibrationsfeedback (Vibration bei richtig oder falsch ausgeführten Übungen)**	○	○	○	○	○
**Musik passend zu den Übungen**	○	○	○	○	○

PF03

Welches digitale Hilfsmittel fänden Sie noch hilfreich?

Bitte geben Sie Ihre Antwort hier ein:

Umgang mit Skoliose

In diesem Teil möchten wir gerne herausfinden wie Sie mit dem Thema Skoliose persönlich umgehen. Bitte beantworten Sie die Fragen so, als würden Sie sich momentan in Skoliosebehandlung befinden.

PG01

Wem haben Sie von Ihrer Skoliose erzählt? *

Bitte wählen Sie alle zutreffenden Antworten aus:

- [ ] Enge Familie (Eltern, Geschwister etc.)
- [ ] Familie (Cousins, Onkel, Tanten, Großeltern etc.)
- [ ] Freund, Freundin, Partner/in
- [ ] Freunde
- [ ] Mitschüler, Arbeitskollegen, Kommilitonen etc.
- [ ] niemandem
- [ ] Sonstiges: ____________

Mehrfachauswahl möglich

PG02

Wie fühlen Sie sich mit Ihrer Skoliose? *

Bitte wählen Sie nur eine der folgenden Antworten aus:

- ○ sehr unwohl
- ○ eher unwohl
- ○ weder noch
- ○ eher wohl
- ○ sehr wohl

Allgemeine Daten

In diesem letzten Teil benötigen wir nur noch einige nicht personenbezogene Daten von Ihnen. Bitte geben Sie auch diese Angaben so an, als würden Sie sich momentan in Skoliosebehandlung befinden.

PH01

Welchem Geschlecht gehören Sie an? *

Bitte wählen Sie nur eine der folgenden Antworten aus:

- ○ weiblich
- ○ männlich
- ○ andere

PH02

Wie ist/war Ihr Körpergewicht in Kilogramm zum Zeitpunkt der Therapie?

Bitte geben Sie Ihre Antwort hier ein:

Sie müssen diese Frage nicht beantworten, wenn Sie nicht möchten. Die Angabe ist allerdings hilfreich für uns.

PH03

Was würden Sie uns gerne noch mitteilen?

Bitte geben Sie Ihre Antwort hier ein:

Übermittlung Ihres ausgefüllten Fragebogens:
Vielen Dank für die Beantwortung des Fragebogens.
