# Supplementary material for: Improving Home-Based Scoliosis Therapy: Findings From a Web-Based Survey
Source: JMIR Rehabil Assist Technol. 2023 Aug 4;10:e46217. doi: 10.2196/46217 (PMC10439467; doi:10.2196/46217)
Supplement: Multimedia Appendix 2 [file rehab_v10i1e46217_app2.pdf]

Nutzerbefragung zum Therapieverhalten von Physiotherapeuten in der Skoliosebehandlung

Im Rahmen eines Forschungsprojektes erforscht das Fraunhofer Institut für Werkzeugmaschinen und Umformtechnik (IWU) in Dresden aktuell die Entwicklung eines Heimtherapie Ansatzes für Skoliose-Patienten. Dazu werden zunächst Nutzerbefragungen bei Physiotherapeuten durchgeführt, die im Bereich der therapeutischen Skoliosebehandlung tätig sind.
Ziel dieses Fragebogens ist es Einblicke in die Erfahrungen von Physiotherapeuten in Bezug auf Therapiemethoden, der Interaktion mit verschiedenen Patientengruppen, der Heimtherapie und den eingesetzten Hilfsmitteln, sowie der Einstellung zu digitalen Hilfsmitteln zu bekommen.
Die in diesem Fragebogen erhobenen Daten sind nicht personenbezogen und die Auswertung aller gegebenen Antworten erfolgt anonym. Wir bedanken uns herzlich für Ihre wertvolle Zeit und Ihren wichtigen Beitrag zu diesem Thema.
Die Beantwortung des Fragebogens dauert maximal 15 Minuten.

In dieser Umfrage sind 36 Fragen enthalten.

Patientengruppen und Therapiemethoden

In diesem ersten Abschnitt wollen wir herausfinden, aus welchen Gruppen sich Ihr Patientenstamm zusammensetzt und welche Therapiemethoden Sie verwenden.

TA01

Bitte wählen Sie aus den folgenden Altersgruppen diejenigen aus, die unter Ihren Patienten am stärksten vertreten sind. *

❗ Bitte wählen Sie die zutreffenden Antworten aus:
Bitte wählen Sie alle zutreffenden Antworten aus:

- ☐ unter 10 Jahre
- ☐ 10 - 14 Jahre
- ☐ 15 - 18 Jahre
- ☐ 18 - 25 Jahre
- ☐ 26 - 30 Jahre
- ☐ 31 - 50 Jahre
- ☐ über 50 Jahre

Mehrfachauswahl möglich. Bitte wählen Sie maximal drei Gruppen aus.

TA02

Bitte geben Sie an wie stark die folgenden Cobb-Winkel-Bereiche bei Ihren Patienten vertreten sind. *

Bitte wählen Sie die zutreffende Antwort für jeden Punkt aus:

	sehr schwach	eher schwach	weder noch	eher stark	sehr stark	gar nicht
**0 - 10°**	○	○	○	○	○	○
**11 - 20°**	○	○	○	○	○	○
**21 - 30°**	○	○	○	○	○	○

TA03

Behandeln Sie weitere Cobb-Winkel-Bereiche? Wenn ja, welche?

Bitte geben Sie Ihre Antwort hier ein:

TA04

Welche der folgenden Therapiemethoden wenden Sie an? *

❗ Bitte wählen Sie die zutreffenden Antworten aus:
Bitte wählen Sie alle zutreffenden Antworten aus:

- ☐ Side Shift Program
- ☐ Schroth
- ☐ FITS
- ☐ DoboMed
- ☐ SEAS
- ☐ Barcelona Scoliosis Physical Therapy School (BSPTS)
- ☐ Spiraldynamik
- ☐ Andere:

Mehrfachauswahl möglich

TA05

Wie beurteilen Sie die Effektivität der genannten Therapiemethoden. *

Bitte wählen Sie die zutreffende Antwort für jeden Punkt aus:

	überhaupt nicht effektiv	eher nicht effektiv	weder noch	eher effektiv	sehr effektiv	kenne ich nicht oder weiß ich nicht
**Schroth**	◯	◯	◯	◯	◯	◯
**Barcelona Scoliosis Physical Therapy School (BSPTS)**	◯	◯	◯	◯	◯	◯
**DoboMed**	◯	◯	◯	◯	◯	◯
**SEAS**	◯	◯	◯	◯	◯	◯
**FITS**	◯	◯	◯	◯	◯	◯
**Side Shift Program**	◯	◯	◯	◯	◯	◯
**Spiraldynamik**	◯	◯	◯	◯	◯	◯

TA06

Bitte beurteilen Sie wie aussagekräftig die folgenden Parameter für die Messung des Therapieerfolgs sind. *

Bitte wählen Sie die zutreffende Antwort für jeden Punkt aus:

	**überhaupt nicht aussagekräftig**	**eher nicht aussagekräftig**	**weder noch**	**eher aussagekräftig**	**sehr aussagekräftig**
**Veränderung des Cobb-Winkel**	○	○	○	○	○
**äußerliche Veränderung (Haltungsverbesserung, sichtbarer Muskelaufbau etc.)**	○	○	○	○	○
**Übungsausführung**	○	○	○	○	○
**Atemkapazität**	○	○	○	○	○

TA07

Welcher Parameter ist für die Messung des Therapieerfolgs Ihrer Meinung nach noch wichtig?

Bitte geben Sie Ihre Antwort hier ein:

TA08

Bitte beurteilen Sie wie die folgenden Altersgruppen Ihrer Patienten auf die Behandlung ansprechen. *

Bitte wählen Sie die zutreffende Antwort für jeden Punkt aus:

	sehr schlecht	eher schlecht	weder noch	eher gut	sehr gut	behandle ich nicht oder weiß ich nicht
**unter 10 Jahre**	◯	◯	◯	◯	◯	◯
**10 - 14 Jahre**	◯	◯	◯	◯	◯	◯
**15 - 18 Jahre**	◯	◯	◯	◯	◯	◯
**19 - 25 Jahre**	◯	◯	◯	◯	◯	◯
**26 - 30 Jahre**	◯	◯	◯	◯	◯	◯
**31 - 50 Jahre**	◯	◯	◯	◯	◯	◯
**Über 50 Jahre**	◯	◯	◯	◯	◯	◯

Therapie in der Praxis

In diesem Teil wollen wir herausfinden, wie Sie die Skoliose-Therapie in Ihrer Praxis bzw. Einrichtung gestalten und wovon Ihre Behandlungsmuster für einzelne Patientengruppen abhängen.

TB01

Wie lang sind die Patienten bei Ihnen in der Regel in Behandlung? *

❗ Bitte wählen Sie eine der folgenden Antworten:
Bitte wählen Sie nur eine der folgenden Antworten aus:

- ◯ 2 - 5 Wochen
- ◯ 6 - 12 Wochen
- ◯ 3 - 6 Monate
- ◯ 7 - 12 Monate
- ◯ länger als 1 Jahr
- ◯ länger als 2 Jahre
- ◯ Andere

TB02

Wie oft sind die Patienten in der Regel bei Ihnen zur Behandlung? *

❗ Bitte wählen Sie eine der folgenden Antworten:
Bitte wählen Sie nur eine der folgenden Antworten aus:

- ◯ mehrmals pro Woche
- ◯ 1 Mal pro Woche
- ◯ mehrmals im Monat
- ◯ 1 Mal im Monat
- ◯ 4 - 5 Mal im Jahr
- ◯ weniger
- ◯ Andere

TB03

Von welchen Faktoren hängt die Häufigkeit der Therapiesitzungen ab? *

❗ Bitte wählen Sie die zutreffenden Antworten aus:
Bitte wählen Sie alle zutreffenden Antworten aus:

- [ ] Cobb-Winkel
- [ ] Alter
- [ ] Vitalkapazität
- [ ] keiner der genannten Faktoren
- [ ] Andere: ____________

Mehrfachauswahl möglich

TB04

Wie lang dauert eine Therapiesitzung bei Ihnen in der Regel? *

❗ Bitte wählen Sie eine der folgenden Antworten:
Bitte wählen Sie nur eine der folgenden Antworten aus:

- ○ 0 - 15 Minuten
- ○ 16 - 30 Minuten
- ○ 31 - 45 Minuten
- ○ 46 - 60 Minuten
- ○ mehr als 60 Minuten
- ○ Sonstiges ____________

TB05

Ab wann stellt sich in der Regel ein merklicher Therapieerfolg ein? *

❗ Bitte wählen Sie eine der folgenden Antworten:
Bitte wählen Sie nur eine der folgenden Antworten aus:

- ◯ 2 - 5 Wochen
- ◯ 6 - 12 Wochen
- ◯ 3 - 6 Monate
- ◯ 7 - 12 Monate
- ◯ länger als 1 Jahr
- ◯ länger als 2 Jahre
- ◯ Andere

TB06

Bitte beurteilen Sie, wie motiviert die genannten Altersgruppen Ihrer Patienten für die Therapie in Ihrer Praxis bzw. Einrichtung sind. *

Bitte wählen Sie die zutreffende Antwort für jeden Punkt aus:

	überhaupt nicht motiviert	eher nicht motiviert	weder noch	eher motiviert	sehr motiviert	behandle ich nicht oder weiß ich nicht
**unter 10 Jahre**	◯	◯	◯	◯	◯	◯
**10 - 14 Jahre**	◯	◯	◯	◯	◯	◯
**15 - 18 Jahre**	◯	◯	◯	◯	◯	◯
**19 - 25 Jahre**	◯	◯	◯	◯	◯	◯
**26 - 30 Jahre**	◯	◯	◯	◯	◯	◯
**31 - 50 Jahre**	◯	◯	◯	◯	◯	◯
**über 50 Jahre**	◯	◯	◯	◯	◯	◯

Therapie zu Hause

In diesem Teil wollen wir herausfinden, welche Erfahrungen Sie mit Therapie-Übungen zu Hause haben.

TC01

Wie oft empfehlen Sie Ihren Patienten in der Regel zur Physiotherapie zusätzliche Übungseinheiten zu Hause? *

❗ Bitte wählen Sie eine der folgenden Antworten:

Bitte wählen Sie nur eine der folgenden Antworten aus:

- ◯ weniger als 1 Mal pro Woche
- ◯ 1 Mal pro Woche
- ◯ 2 Mal pro Woche
- ◯ 3 Mal pro Woche
- ◯ 4 Mal pro Woche
- ◯ 5 Mal pro Woche
- ◯ mehr als 5 Mal pro Woche
- ◯ Andere ________

TC02

Bitte geben Sie an, inwiefern Ihre Empfehlung für die Heimtherapie von den folgenden Faktoren auf Patientenseite abhängt. *

Bitte wählen Sie die zutreffende Antwort für jeden Punkt aus:

	**trifft überhaupt nicht zu**	**trifft eher nicht zu**	**weder noch**	**trifft eher zu**	**trifft voll zu**
**Cobb-Winkel**	◯	◯	◯	◯	◯
**Alter**	◯	◯	◯	◯	◯
**Persönliche Motivation**	◯	◯	◯	◯	◯
**Kognitive Eignung**	◯	◯	◯	◯	◯

TC03

Welche Faktoren sind noch wichtig für Ihre Empfehlung für die Heimtherapie?

Bitte geben Sie Ihre Antwort hier ein:

TC04

Bitte beurteilen Sie, wie motiviert die folgenden Altersgruppen Ihren Patienten für die Heimtherapie sind. *

Bitte wählen Sie die zutreffende Antwort für jeden Punkt aus:

	überhaupt nicht motiviert	eher nicht motiviert	weder noch	eher motiviert	sehr motiviert	behandle ich nicht oder weiß ich nicht
**unter 10 Jahre**	○	○	○	○	○	○
**10 - 14 Jahre**	○	○	○	○	○	○
**15 - 18 Jahre**	○	○	○	○	○	○
**19 - 25 Jahre**	○	○	○	○	○	○
**26 - 30 Jahre**	○	○	○	○	○	○
**31 - 50 Jahre**	○	○	○	○	○	○
**Über 50 Jahre**	○	○	○	○	○	○

TC05

Aus welchen Therapiemethoden empfehlen Sie Übungen für zu Hause? *

❗ Bitte wählen Sie die zutreffenden Antworten aus:
Bitte wählen Sie alle zutreffenden Antworten aus:

- ☐ Schroth
- ☐ Barcelona Scoliosis Physical Therapy School (BSPTS)
- ☐ DoboMed
- ☐ SEAS
- ☐ FITS
- ☐ Side Shift Program
- ☐ Spiraldynamik
- ☐ keine
- ☐ Andere: ____________

Mehrfachauswahl möglich

Kommunikation mit Patienten

In diesem Teil wollen wir herausfinden, wie Sie mit Ihren Patienten kommunizieren und wie effektiv sie die Kommunikation mit ihnen in Bezug auf verschiedene Aspekte einschätzen.

TD01

Wie schätzen Sie die Effektivität der Kommunikation mit Ihren Patienten allgemein ein? *

❗ Bitte wählen Sie eine der folgenden Antworten:
Bitte wählen Sie nur eine der folgenden Antworten aus:

- ◯ überhaupt nicht effektiv
- ◯ eher nicht effektiv
- ◯ weder noch
- ◯ eher effektiv
- ◯ sehr effektiv

TD02

Wie schnell verstehen Patienten in der Regel Ihre Übungsanweisungen? *

❗ Bitte wählen Sie eine der folgenden Antworten:
Bitte wählen Sie nur eine der folgenden Antworten aus:

- ◯ sehr langsam
- ◯ eher langsam
- ◯ weder noch
- ◯ eher schnell
- ◯ sehr schnell

TD03

Wie sehr sind die folgenden Aspekte auf Patientenseite Ihrer Meinung nach Gründe für mögliche Kommunikationsschwierigkeiten (Missverständnisse etc.)? *

Bitte wählen Sie die zutreffende Antwort für jeden Punkt aus:

	trifft überhaupt nicht zu	trifft eher nicht zu	weder noch	trifft eher zu	trifft voll zu
**komplizierter Übungsablauf**	○	○	○	○	○
**Wissensstand über Skoliose**	○	○	○	○	○
**Bereitschaft der Auseinandersetzung mit Skoliose**	○	○	○	○	○
**Motivation für die Therapie**	○	○	○	○	○
**Nachvollziehbarkeit der Sinnhaftigkeit bestimmter Übungen**	○	○	○	○	○
**Nachvollziehbarkeit der Dauer der Sitzungen**	○	○	○	○	○
**Nachvollziehbarkeit der Therapiedauer**	○	○	○	○	○

Geräte als Hilfsmittel

In diesem Abschnitt wollen wir herausfinden, welche Geräte Sie in Ihrer Therapie nutzen und wie hilfreich Sie welche davon einschätzen.

TE01

Welche der folgenden Geräte nutzen Sie für die Therapie in Ihrer Praxis und welche empfehlen Sie Ihren Patienten für ihre Übungen zu Hause? *

Bitte wählen Sie die zutreffende Antwort für jeden Punkt aus:

	in der Praxis	zu Hause	beides	gar nicht
**Polstermatten oder Gymnastikmatten**	○	○	○	○
**Unterlagen (Schaumstoffrollen etc.)**	○	○	○	○
**Sandsäckchen oder Reisssäckchen**	○	○	○	○
**Kissen**	○	○	○	○
**Sprossenwand**	○	○	○	○
**Spiegel**	○	○	○	○
**lange Stäbe**	○	○	○	○
**Hocker oder Stühle**	○	○	○	○
**Tische**	○	○	○	○
**Gymnastikball oder Medizinball**	○	○	○	○
**Balance Board**	○	○	○	○
**Bänder (Gummibänder, Therabänder etc.)**	○	○	○	○

TE02

Welche Geräte nutzen Sie noch als Hilfsmittel oder empfehlen Ihren Patienten für die Heimtherapie?

Bitte geben Sie Ihre Antwort hier ein:

TE03

Bitte beurteilen Sie, wie hilfreich die genannten Hilfsmittel für die Skoliose-Therapie sind. *

Bitte wählen Sie die zutreffende Antwort für jeden Punkt aus:

	überhaupt nicht hilfreich	eher nicht hilfreich	weder noch	eher hilfreich	sehr hilfreich	nutze ich nicht oder weiß ich nicht
**Polstermatten oder Gymnastikmatten**	◯	◯	◯	◯	◯	◯
**Unterlagen (Schaumstoffrollen etc.)**	◯	◯	◯	◯	◯	◯
**Sandsäckchen oder Reisssäckchen**	◯	◯	◯	◯	◯	◯
**Kissen**	◯	◯	◯	◯	◯	◯
**Sprossenwand**	◯	◯	◯	◯	◯	◯
**Spiegel**	◯	◯	◯	◯	◯	◯
**lange Stäbe**	◯	◯	◯	◯	◯	◯
**Hocker oder Stühle**	◯	◯	◯	◯	◯	◯
**Tische**	◯	◯	◯	◯	◯	◯
**Gymnastikball oder Medizinball**	◯	◯	◯	◯	◯	◯
**Balance Board**	◯	◯	◯	◯	◯	◯
**Bänder (Gummibänder, Therabänder etc.)**	◯	◯	◯	◯	◯	◯

Digitale Hilfsmittel

In diesem Teil wollen wir herausfinden, wie Sie zu digitalen Hilfsmitteln in der Skoliose-Therapie stehen und welche davon Sie bereits wie verwenden.

TF01

Welche digitalen Hilfsmittel nutzen Sie bereits in der Skoliose-Therapie? *

❗ Bitte wählen Sie die zutreffenden Antworten aus:
Bitte wählen Sie alle zutreffenden Antworten aus:

- ☐ Computer
- ☐ Smartphone
- ☐ Tablet
- ☐ Uhr (digital oder auch analog)
- ☐ keine
- ☐ Andere: ______

Mehrfachauswahl möglich

TF02

Wozu nutzen Sie digitale Hilfsmittel in der Skoliose-Therapie? *

❗ Bitte wählen Sie die zutreffenden Antworten aus:
Bitte wählen Sie alle zutreffenden Antworten aus:

- ☐ Dokumentation des Therapieerfolgs
- ☐ Kommunikation mit den Patienten
- ☐ Übungsanleitungen
- ☐ gar nicht
- ☐ Andere: ______

Mehrfachauswahl möglich

TF03

Bitte geben Sie an wie sinnvoll Sie den verstärkten Einsatz von digitalen Hilfsmitteln für die folgenden Anwendungen fänden. *

Bitte wählen Sie die zutreffende Antwort für jeden Punkt aus:

	überhaupt nicht sinnvoll	eher nicht sinnvoll	weder noch	eher sinnvoll	sehr sinnvoll
**Beobachtung des Therapiefortschritts**	○	○	○	○	○
**Kommunikation mit den Patienten**	○	○	○	○	○
**Verbessung der Heimtherapie**	○	○	○	○	○
**Steigerung der Patientenmotivation**	○	○	○	○	○
**Virtuelle Therapiesitzungen mit Patienten**	○	○	○	○	○
**Feedback zur Heimtherapie aus der Praxis**	○	○	○	○	○
**Übungsanleitungen**	○	○	○	○	○

TF04

Für welche Anwendung können Sie sich den verstärkten Einsatz digitaler Hilfsmittel in der Heimtherapie noch vorstellen?

Bitte geben Sie Ihre Antwort hier ein:

TF05

Welche Parameter müssten Ihrer Meinung nach für eine Beobachtung bzw. Verbesserung der Heimtherapie gemessen werden? *

❶ Bitte wählen Sie die zutreffenden Antworten aus:
Bitte wählen Sie alle zutreffenden Antworten aus:

- ☐ Vitalkapazität (Puls etc.)
- ☐ Position und Bewegung von bestimmten Körperteilen (z.B. Extremitäten)
- ☐ Atemkapazität
- ☐ keine
- ☐ Andere: ____________

Mehrfachauswahl möglich

TF06

Welche Parameter müssten bei einem Initialtraining für die Kalibrierung eines digitalen Hilfsmittels angegeben bzw. gemessen werden? *

❶ Bitte wählen Sie die zutreffenden Antworten aus:
Bitte wählen Sie alle zutreffenden Antworten aus:

- ☐ Vitalkapazität (Puls etc.)
- ☐ Atemkapazität
- ☐ Cobb-Winkel
- ☐ Alter
- ☐ Körpergewicht
- ☐ Körpergröße
- ☐ Sensorische Aufzeichnung der Übungsausführung
- ☐ Sonstiges: ____________

Mehrfachauswahl möglich

TF07

Welche Art von Daten können Sie sich für den Austausch mit Patienten zum Therapie-Monitoring vorstellen? *

❗ Bitte wählen Sie die zutreffenden Antworten aus:
Bitte wählen Sie alle zutreffenden Antworten aus:

- [ ] Übungsanleitungen als Bilder
- [ ] Übungsanleitungen als Videos
- [ ] Übungsanleitungen als Sprachaufnahme
- [ ] Übungsaufzeichnung als Bild
- [ ] Übungsaufzeichnung als Video
- [ ] Sensordaten zur Position und Bewegung von Körperteilen
- [ ] Sensordaten zur Dehnung an bestimmten Körperstellen
- [ ] Sensordaten zur Muskelaktivität an bestimmten Körperstellen
- [ ] Zeitdaten zur Übungsdauer
- [ ] Andere: ____________________

Mehrfachauswahl möglich

Allgemeine Fragen

In diesem letzten Teil haben wir noch ein paar abschließende allgemeine Fragen für Sie.

TG01

Bitte wählen Sie aus dem folgenden Bild diejenigen Hilfsmittel aus, die Sie für ein effektives Therapie-Gerät beibehalten würden und geben Sie an welche(s) Sie ggf. hinzufügen würden.

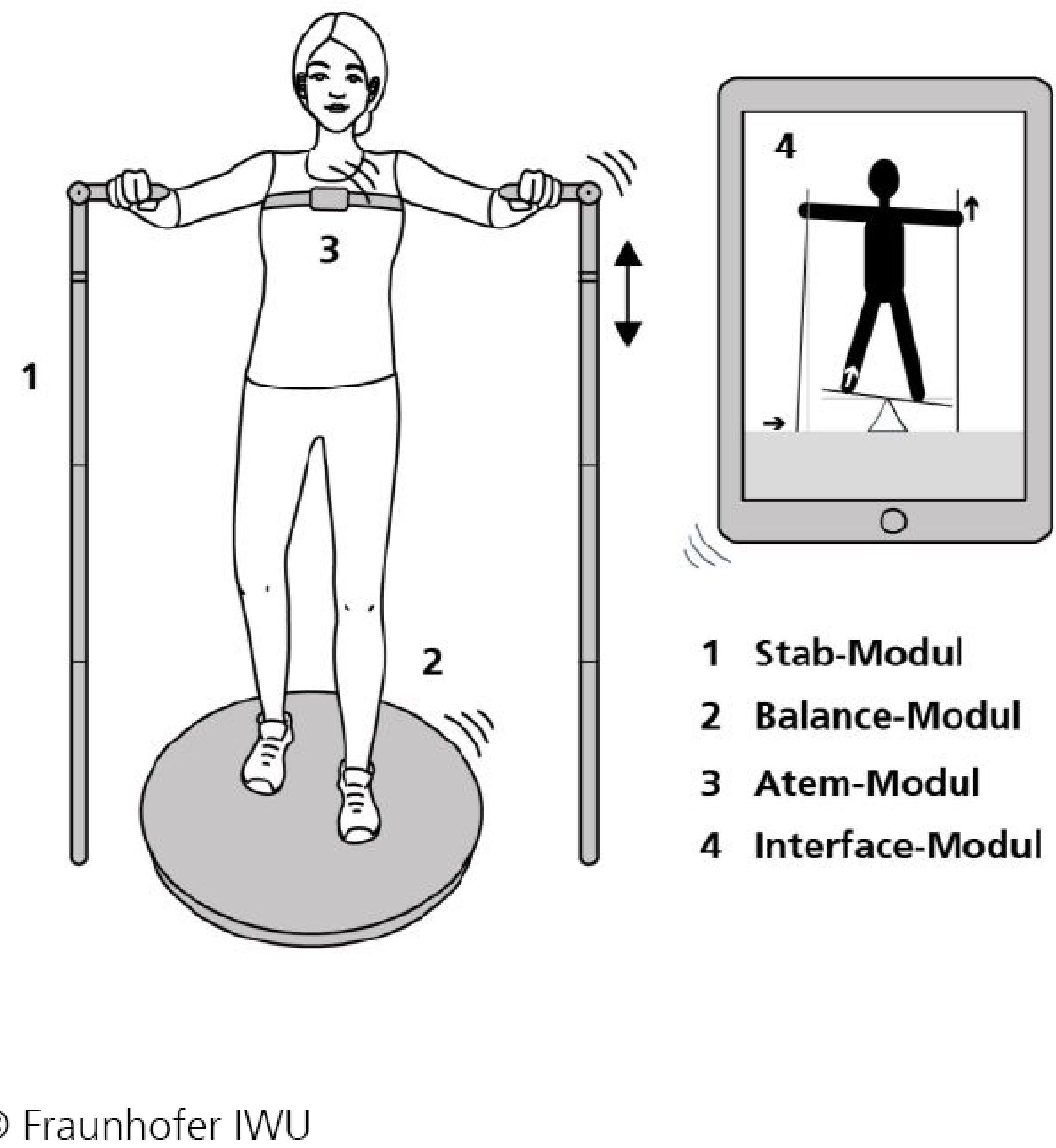

*

❗ Bitte wählen Sie die zutreffenden Antworten aus:
Bitte wählen Sie alle zutreffenden Antworten aus:

- ☐ Stab-Modul
- ☐ Balance-Modul
- ☐ Atem-Modul
- ☐ Interface-Modul
- ☐ Hinzufügen: ____

SIe können auch mehrere Hilfsmittel hinzufügen. Bitte trennen Sie sie dann mit einem Komma.

TG02

Wie sinnvoll fänden Sie einen spielerischen Ansatz für die Skoliose-Therapie? (zum Beispiel Belohnungen für richtig ausgeführte Übungen oder für das regelmäßige Üben) *

❗ Bitte wählen Sie eine der folgenden Antworten:
Bitte wählen Sie nur eine der folgenden Antworten aus:

- ◯ überhaupt nicht sinnvoll
- ◯ eher nicht sinnvoll
- ◯ weder noch
- ◯ eher sinnvoll
- ◯ sehr sinnvoll

TG03

Wie sinnvoll fänden Sie es, wenn Patienten sich beim Therapieerfolg miteinander vergleichen könnten? ("Skoliose-Strava" oder "Skoliose-Facebook") *

❗ Bitte wählen Sie eine der folgenden Antworten:
Bitte wählen Sie nur eine der folgenden Antworten aus:

- ◯ überhaupt nicht sinnvoll
- ◯ eher nicht sinnvoll
- ◯ weder noch
- ◯ eher sinnvoll
- ◯ sehr sinnvoll

TG04

Was würden Sie uns gerne noch mitteilen?

Bitte geben Sie Ihre Antwort hier ein:

Übermittlung Ihres ausgefüllten Fragebogens:
Vielen Dank für die Beantwortung des Fragebogens.
